# A Knowledge Query Network Model Based on Rasch Model Embedding for Personalized Online Learning

**DOI:** 10.3389/fpsyg.2022.846621

**Published:** 2022-08-01

**Authors:** Yan Cheng, Gang Wu, Haifeng Zou, Pin Luo, Zhuang Cai

**Affiliations:** ^1^School of Computer and Information Engineering, Jiangxi Normal University, Nanchang, China; ^2^Jiangxi Provincial Key Laboratory of Intelligent Education, Nanchang, China

**Keywords:** personalized education, deep learning, knowledge tracking, forgetting behavior, interpretability

## Abstract

The vigorous development of online education has produced massive amounts of education data. How to mine and analyze education big data has become an urgent problem in the field of education and big data knowledge engineering. As for the dynamic learning data, knowledge tracing aims to track learners’ knowledge status over time by analyzing the learners’ exercise data, so as to predict their performance in the next time step. Deep learning knowledge tracking performs well, but they mainly model the knowledge components while ignoring the personalized information of questions and learners, and provide limited interpretability in the interaction between learners’ knowledge status and questions. A context-aware attentive knowledge query network (CAKQN) model is proposed in this paper, which combines flexible neural network models with interpretable model components inspired by psychometric theory. We use the Rasch model to regularize the embedding of questions and learners’ interaction tuples, and obtain personalized representations from them. In addition, the long-term short-term memory network and monotonic attention mechanism are used to mine the contextual information of learner interaction sequences and question sequences. It can not only retain the ability to model sequences, but also use the monotonic attention mechanism with exponential decay term to extract the hidden forgetting behavior and other characteristics of learners in the learning process. Finally, the vector dot product is used to simulate the interaction between the learners’ knowledge state and questions to improve the interpretability. A series of experimental results on 4 real-world online learning datasets show that CAKQN has the best performance, and its AUC value is improved by an average of 2.945% compared with the existing optimal model. Furthermore, the CAKQN proposed in this paper can not only track learners’ knowledge status like other models but also model learners’ forgetting behavior. In the future, our research will have high application value in the realization of personalized learning strategies, teaching interventions, and resource recommendations for intelligent online education platforms.

## Introduction

With the rapid development of Internet technology and artificial intelligence technology in the field of education, online learning platforms such as massive open online courses (MOOCs) have become increasingly popular. Learners’ activities on online learning platforms have generated massive amounts of educational data. How to mine and analyze large amounts of educational data has become an urgent problem in the field of education and big data knowledge engineering (Hu et al., 2020). Since learners’ behavior, knowledge state, and psychological factors in the learning process are the key factors for evaluating their learning effectiveness (Yang and Li, 2018), and these factors are constantly changing over time, it is of great significance to construct a learner model oriented to dynamic learning data.

**FIGURE 1:**
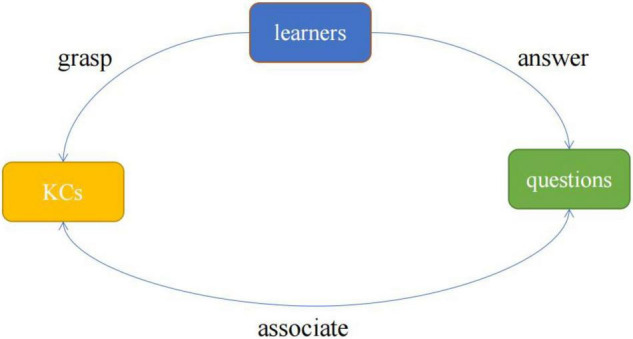
The relationship between learners, questions, and KC.

Different from the cognitive diagnosis model (CDM) for static learning data, knowledge tracing (KT) aims to dynamically track learners’ knowledge status over time by analyzing the learners’ historical exercise data, so as to predict their performance in the next time step. The learner’s historical exercise data is a sequence composed of the questions, the knowledge components (KCs) contained in the questions, and the learner’s answers (Liu et al., 2021). The three core elements of questions, KCs and learners constitute the three basic objects of the KT data processing, the interaction between them is shown in Figure 1. KT is the quantitative analysis and modeling of the relationship between three types of objects. For example, the prediction of students’ knowledge mastery state is to calculate the mastery probability between “students and knowledge” by using the interaction between “students and problems” and the correlation information between “problems and knowledge.” The interaction between different objects is the main information used in the KT modeling process (Sun et al., 2021). Therefore, the KT model not only needs to accurately assess the learner’s knowledge state and predict their answer in the future but also needs to provide explanations for the interaction between different objects (Hu et al., 2020).

Traditional KT methods mainly include Bayesian knowledge tracking (BKT) (Corbett and Anderson, 1994) based on hidden Markov model (HMM) (Rabiner and Juang, 1986) and item response theory (IRT) (Fan, 1998). In recent years, researchers have tended to use more complex and flexible models like deep networks to make full use of hidden information in large-scale learner response datasets. The deep knowledge tracing (DKT) (Piech et al., 2015) model introduced recurrent neural network (RNN) into the KT field for the first time and achieved success. Compared with the traditional KT model, the predictive ability based on the deep learning method has been significantly improved. However, most of the current KT methods based on deep learning mostly use KCs to index questions, ignoring the rich information contained in the questions and the context. For example, investigating different questions of the same KC may cause individual differences between questions due to different difficulty settings. In addition, the personalized interaction between learners’ knowledge status and questions representation is often overlooked, which leads to poor interpretability of the KT method based on deep learning. In response to the above problems, we propose a context-aware attentive knowledge query network (CAKQN) model based on the embedded Rasch model, which is the single parameter IRT model. First, input the learner interaction tuple and questions into the embedded component based on the Rasch model to obtain personalized representations of the learner interaction tuple and questions, and capture the characteristics of individual differences between different questions containing the same KC and the learners’ personal abilities. Next, based on the definition of memory trace decline in educational psychology theory (Bailey, 1989) that human memory fades automatically over time, a network structure of long short-term memory network + monotonic attention mechanism is designed to learn personalized learner knowledge state and context-aware representation of the questions. The learning process of learners is continuous, so the sequence structure of learning records cannot be destroyed in the KT modeling process. The structure we designed uses a monotonic attention mechanism with an exponential decay term to reduce the importance of learner interaction tuples in the distant past without destroying the sequence structure of the learners’ historical learning records, and it can extract features such as forgetting behavior that exist in the learning process of learners. Finally, based on the fact that learners answer questions based on their knowledge status and personal abilities, the vector dot product is used to simulate the personalized interaction between learners’ knowledge status and questions to improve the interpretability of the model. We used four publicly available real online education datasets to evaluate the model. Experiments show that the CAKQN model has the best performance, and its AUC value is 2.945% higher than the existing optimal model on average. In addition, our paper also conducted a series of ablation analysis and knowledge tracking visualization experiments to verify the excellent interpretability and personalization capabilities of the CAKQN model. In the future, our research will have high application value in the realization of personalized learning strategies, teaching interventions, and resource recommendations for intelligent online education platforms.

## Related Work

### Traditional Knowledge Tracking Methods

Traditional knowledge tracking methods are mainly divided into two categories: IRT and BKT, and IRT is one of the important psychological and educational theories (Cheng et al., 2019). The single-parameter IRT model (i.e., Rasch model) outputs the probability of learners answering the items correctly during the test according to the learner’s ability level and the difficulty level of the items (i.e., questions). The probability is defined by the item response function with the following characteristics: if the learner’s ability level is higher, the learner has a higher probability of answering an item correctly. Conversely, if an item is more difficult, the probability of the learner answering the item correctly is lower. The item response function is defined as follows:


(1) $$P\,\left(a\right)=\sigma \,\left(\theta -\beta_{j}\right)=\frac{1}{1+e^{-D\,\left(\theta -\beta_{j}\right)}}$$

The more complex two-parameter item response function introduces item discrimination α_*j*_, which is defined as follows:


(2) $$P\,\left(a\right)=\sigma \,\left(\theta -\beta_{j}\right)=\frac{1}{1+e^{-D\,\alpha_{j}\,\left(\theta -\beta_{j}\right)}}$$

Where σ is the sigmoid function, *D* is a constant, usually set to 1.7, θ is learner’s ability level, β_*j*_ is the difficulty level of item *j*. Since the IRT model was originally designed for educational testing environments, the model assumes that learners’ abilities remain unchanged during the testing process. In reality, the knowledge state of learners changes with time step, so it cannot be directly applied to KT tasks.

The BKT model updates the learner’s knowledge state through HMM modeling, and predicts the learner’s performance at the next time step accordingly. However, many simplified assumptions used in the BKT model are impractical. One of them is that all learners and questions containing the same KC are considered the same. Therefore, the researchers studied various personalizations of the BKT model. Some researchers endow the BKT model with personalized capabilities on specific parameters of KC (Pardos and Heffernan, 2011) and specific parameters of learners (Yudelson et al., 2013). Some other researchers have also studied the synthesis of the BKT model and the IRT model (Khajah et al., 2014; Wilson et al., 2016) to enhance the model’s personalization ability when dealing with questions and learners. However, such expansion usually requires a lot of feature engineering work and will result in a significant increase in computing requirements.

### Deep Learning Knowledge Tracking

In recent years, deep learning has attracted attention from researchers with its powerful feature extraction capabilities. Many researchers have applied it to the KT field, which is called DLKT (deep learning knowledge tracing) (Liu et al., 2021). Compared with BKT and IRT, DLKT does not require manually annotated KC information and can capture more complex learner knowledge representations from large-scale learner response datasets. DKT and dynamic key-value memory network (DKVMN) (Zhang et al., 2017) have shown strong predictive ability in predicting learners’ future performance, and have become the benchmark for subsequent DLKT methods. DKT takes the learner’s historical learning interaction sequence as input, then uses RNN to encode it into the learner’s knowledge state, and finally inputs it into a linear layer activated by a Sigmoid function to get the prediction result. DKT, which simply represents the learner’s knowledge state as a vector, while DKVMN uses a static external matrix to store KC and uses a dynamic matrix to update the learner’s mastery of KC. However, the simple splicing between the two vectors representing the learner’s knowledge state and KC in the DKVMN model is not enough to explain the process of interaction between the learner’s knowledge state and the KC contained in the question (Daniluk et al., 2017). The knowledge query network (KQN) (Lee and Yeung, 2019) model uses the vector dot product to more accurately simulate the interaction between the learner’s knowledge state and KC, and achieves better results. Self-attentive knowledge tracing (SAKT) (Pandey and Karypis, 2019) model is the first to use the Transformer structure in the KT field to replace RNN to automatically focus on the record of questions in the learner’s historical interaction sequence that has a greater impact on the prediction results and achieves model performance. The substantial increase. However, the above models use KCs to index questions, that is, all different questions containing the same KC are regarded as equivalent. This way ignores the rich information contained in the question itself and the context. Context-aware attention knowledge tracing (AKT) (Ghosh et al., 2020). The framework based on the SAKT model uses the Rasch model to regularize concept and question embeddings. These embeddings can capture questions that contain the same KC, without using too many parameters. In addition, AKT also uses a new monotonic attention mechanism to link learners’ future responses to questions with their historical interaction sequences to extract features such as hidden forgetting behavior in the learning process of learners. However, the AKT model also uses unreasonable vector simple splicing to simulate learner knowledge status and question interaction, and it loses the ability to model sequence due to the Transformer structure like SAKT.

Considering the advantages and disadvantages of KQN model and AKT model, this paper proposes a context-aware knowledge query network (CAKQN) based on Rasch model embedding. It not only retains the ability of model sequence but also obtains personalized contextual representations of questions and learners. We improve the model’s performance in predicting future learner responses. Moreover, the interpretability of the model in terms of learner knowledge status and questions interaction is enhanced.

## Our Proposed Method

This section first introduces the problem setup of knowledge tracing and the symbolic representation of related concepts, then introduces the difference between ordinary attention mechanism and monotonic attention mechanism with exponential decay, and then describes the overall context-aware knowledge query network model based on Rasch model embedding framework, and finally introduce each component of the model and its loss function in turn.

### Knowledge Tracing Problem Setup

Assuming that there are M questions and N KCs in the original dataset, each learner’s interaction record is composed of the learner’s long questions and responses at each time step. For the learner *i* at time step *t*, a learner interaction tuple $x_{t}=\left(q_{t}^{i},c_{t}^{i},r_{t}^{i}\right)$ is composed of: the question $q_{t}^{i}$ he or she answered, the KC $c_{t}^{i}$ covered by the question, and the learner’s response $r_{t}^{i}$ to the question. Where $q_{t}^{i}$ is the question index, $q_{t}^{i}\in \left\{1,\cdots ,M\right\}$, $c_{t}^{i}$ is the KC index, $c_{t}^{i}\in \left\{1,\cdots ,N\right\}$, and $r_{t}^{i}$ is the response, $r_{t}^{i}=\left\{0,1\right\}$. Under this notation, (*q*_*t*_,*c*_*t*_,1) means learner *i* responded to question *q*_*t*_ on concept *c*_*t*_ correctly at time step *t*. This setting is different from some previous deep knowledge tracking work, which often ignores the question index and set the learner’s interaction tuple as $\left(c_{t}^{i},r_{t}^{i}\right)$. For convenience, the superscript *i* is omitted in the following discussion. Therefore, given learner’s historical learning interaction sequence *X*_*t*_ = {*x*_1_,*x*_2_,⋯,*x*_*t*_} at time step *t* and question *q*_*t+1*_ on concept *c*_*t+1*_ at time step *t+1*, the goal of the KT model is to find the probability *P*(*r*_*t* + 1_ = 1|*X*_*t*_,*q*_*t* + 1_,*c*_*t* + 1_).

### Monotonic Attention Mechanism With Exponential Decay

Under the ordinary dot product attention mechanism, the input is mapped to three vectors: *Query*, *Key*, and *Value* by embedding layer, and values of dimension *D*_*q*_ = *D*_*k*_, *D*_*k*_ and *D*_*v*_. Let *q*_*t*_ ∈ ℝ*^D^*_*^k^*_^×1^ donate the *Query* corresponding at time step *t*, the calculation formula of the scaled dot product attention value α_*t,τ*_ normalized by the softmax function is:


(3) $$\alpha_{t,\tau }=\mathrm{Softmax}\,\left(\frac{q_{t}^{T}\,k_{\tau }}{\sqrt{D_{k}}}\right)=\frac{\exp\,\left(\frac{q_{t}^{T}\,k_{\tau }}{\sqrt{D_{k}}}\right)}{\sum_{\tau^{\prime }}\exp\,\left(\frac{q_{t}^{T}\,k_{\tau }}{\sqrt{D_{k}}}\right)}\in \left[0,1\right]$$

Where *k*_τ_ ∈ ℝ*^D^*_*^k^*_^×1^ donate *Key* at time step τ.

However, this ordinary zoom dot product attention mechanism is not enough for KT tasks. The reason is that learners have forgetting behaviors in the learning process, and learners will have memory decline in the real world (Pashler et al., 2009). In other words, when the model predicts the learner’s reaction to the next question, his performance in the distant past is not as important as his recent performance. Therefore, Ghosh et al. (2020) add a multiplicative exponential decay term to the attention scores. So the calculation of the new monotonic attention mechanism is as follows:


(4) α′t,τ=exp⁢(st,τ)∑τ′exp⁢(st,τ′)

(5) $$s_{t,\tau }=\frac{\exp\,\left(-\theta \cdot d\,\left(t,\tau \right)\right)\cdot q_{t}^{T}\,k_{\tau }}{\sqrt{D_{k}}}$$

Where θ > 0 is a learnable decay rate parameter, and *d*(*t*,τ) is temporal distance measure between time steps *t* and τ. In other words, the attention weight of the current question to the past question not only depends on the similarity between the corresponding sums, but also depends on the relative time steps between them. The calculation method of *d*(*t*,τ) is as follows:


(6) $$d\,\left(t,\tau \right)=\left|t-\tau \right|\cdot \sum_{t^{\prime }=\tau +1}^{t}\gamma \,\left(t,t^{\prime }\right)$$

(7) $$\gamma \,\left(t,t^{\prime }\right)=\frac{\exp\,\left(\frac{q_{t}^{T}\,k_{t^{\prime }}}{\sqrt{D_{k}}}\right)}{\sum_{1\leq \tau^{\prime }\leq t}\exp\,\left(\frac{q_{t}^{T}\,k_{\tau^{\prime }}}{\sqrt{D_{k}}}\right)}$$

The calculation formula of the final output of the monotonic attention mechanism is as follows:


(8) Monotonic⁢_⁢Attention⁢(Query,Key,Value)=∑τ=1tα′t,τ⁢vτ

Where *v*_τ_ ∈ ℝ*^D^*_*^k^*_^×1^ donate *Key* at time step τ.

### Model Framework

**FIGURE 2:**
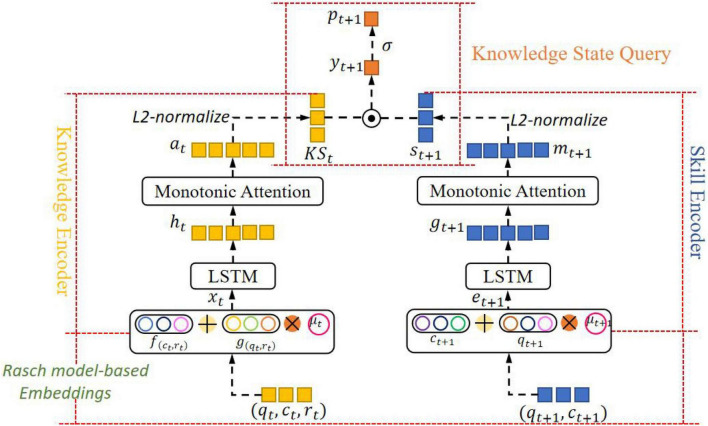
The overall framework of the CAKQN model.

This paper proposes a context-aware knowledge query network based on Rasch model embedding. Figure 2 shows the overall framework of the model. It contains 4 components: *Embedded Layer Based on Rasch Model*, *Knowledge State Encoder*, *Question Encoder*, and *Knowledge Status Query*.

(1) *Embedded Layer Based on Rasch Model*: Get the personalized embedding of the learner interaction tuple at the current time step and the next-time step question, and capture the characteristics of individual differences between different questions on the same KC and the learners’ personal abilities.

(2) *Knowledge State Encoder*: First, use the location information provided by the long short-term memory network to model the context of the learner’s historical interaction sequence, and retain the ability of the model to model the sequence. Then, the monotonic attention mechanism with exponential decay term is used to reduce the importance of learner interaction tuples in the distant past, extract the forgetting behavior and other characteristics of learners in the learning process, and obtain the contextual perception vector of the learner’s knowledge state at the current time step.

(3) *Question Encoder*: It is exactly the same as the network structure adopted by the knowledge state encoder to obtain the context awareness vector of the question at the current time step.

(4) *Knowledge Status Query*: The dot product operation is performed on the vector representing the learner’s knowledge state and the question at the current time step to simulate the interaction between the learner’s knowledge state and the question, and the result of the dot product is input into the sigmoid function to obtain the final prediction of the probability that the learner will answer correctly at the next time step.

#### Embedding Layer Based on Rasch Model

Existing KT methods mostly use KC to index questions, that is, set *q*_*t*_ = *c*_*t*_, because the number of questions in the real world is far greater than the number of KC, so using KC to index questions can effectively avoid over-parameterization and over-fitting. However, this setting ignores the individual differences between question covering the same KC, and limits the flexibility of the KT method and its ability to be personalized.

This article uses the classic Rasch model in psychometric theory to construct learner interaction tuples and question embedding. There are two important parameters in the Rasch model: the difficulty of the question and the ability of the learners. Therefore, at time step *t*, the final embedded representation of the learner’s interaction tuple is expanded to:


(9) $$x_{t}=f_{\left(c_{t},r_{t}\right)}+\mu_{t}\cdot g_{\left(q_{t},r_{t}\right)}$$

Where *f*_(*c*_*t*_,*r*_*t*_)_ ∈ ℝ*^D^*, *g*_(*q*_*t*_,*r*_*t*_)_ ∈ ℝ*^D^*, they respectively, represent the embedding vector of the KC response tuple and the embedding vector of the question response tuple. And μ_*t*_ is a learnable scalar, which represents the learner’s ability parameter. At the next time step, the final embedded representation of the question is expanded to:


(10) $$e_{t+1}=c_{t+1}+\mu_{t+1}\cdot q_{t+1}$$

Where *c*_*t* + 1_ ∈ ℝ*^D^* is the embedding vector of KC contained in this question, *q*_*t* + 1_ ∈ ℝ*^D^* is the embedding vector of the question. And μ_*t+1*_ is also a learnable scalar, it represents the difficulty parameter, which controls the degree of deviation of the question from the KC contained in it. These Rasch model-based embeddings strike an appropriate balance between obtaining personalized representations and avoiding excessive parameterization.

#### Knowledge State Encoder

In the *Knowledge State Encoder*, the structure of the LSTM layer + monotonic attention mechanism layer is used to obtain the context perception results of learner interaction sequences. The way learners understand and learn when answering questions is based on their own knowledge state, and the learner’s knowledge state is related to the learner’s historical learning interaction sequence. For two learners with different historical learning interaction sequences, the way they understand the same question and the knowledge they gain from the exercise may be different. Therefore, we use the LSTM structure to ensure that the original learner history learning interaction sequence is not destroyed on the time scale, and introduce the monotonic attention mechanism to summarize the performance of the past learners in the correct time range, tap the hidden features of the learning process, and then obtain their knowledge state. Given input *x*_*t*_, the knowledge state encoder first inputs it to the LSTM layer to obtain its hidden state *h*_*t*_. Then input *h*_*t*_ to the monotonic attention mechanism layer to get the weighted vector *a*_*t*_, and finally a through a fully connected layer and L2 normalization to get the final output knowledge state vector *KS*_*t*_. The calculation process is as follows:


(11) $$\left\{ \begin{array}{c} i_{t}=\sigma \,\left(W_{i}\,\left[x_{t},h_{t-1},c_{t-1}\right]+b_{i}\right)\;\\ f_{t}=\sigma \,\left(W_{f}\,\left[x_{t},h_{t-1},c_{t-1}\right]+b_{f}\right)\;\\ o_{t}=\sigma \,\left(W_{o}\,\left[x_{t},h_{t-1},c_{t-1}\right]+b_{o}\right)\;\\ c_{t}=f_{t}\,c_{t-1}+i_{t}\,\tanh\left(W_{c}\,\left[x_{t},h_{t-1}\right]+b_{c}\right)\\ h_{t}=o_{t}\,t\,a\,n\,h\,\left(c_{t}\right)\; \end{array}\right.$$

Where *i*_*t*_, *f*_*t*_, *o*_*t*_, *c*_*t*_ are the input gate, forget gate, output gate and unit state, respectively.


(12) $$a_{t}=\mathrm{Monotonic}\,\_\,\mathrm{Attention}\,\left(x_{t},x_{t},h_{t}\right)$$

(13) $${\mathrm{KS}}_{t}=L\,2\,\_\,\mathrm{normalize}\,\left(W_{h,\mathrm{KS}}\,a_{t}+b_{h,\mathrm{KS}}\right)$$

Where *W*_*h*,*KS*_ ∈ ℝ*^d^*^×^*^H^*^_*LSTM*_^, *b*_*h*,*KS*_ ∈ ℝ*^d^*, and *H*_*LSTM*_ is the size of the hidden layer of the LSTM, *d* is the dimension of the knowledge state vector *KS*_*t*_ and the question vector *S*_*t+1*_. *L*2_*normalize* is L2 normalization, the reason for this limitation is to allow the knowledge state vector and the question vector to be a dot product. In addition, in order to avoid overfitting, regularization is used in the output layer of LSTM.

#### Question Encoder

In this article, the question encoder uses the same network structure as the knowledge state encoder, and the purpose is also to capture the context-aware results of the question at the next time step. The specific calculation process of the input question embedding *e*_*t+1*_ to obtain the question vector *s*_*t+1*_ by the question encoder is as follows:


(14) $$\left\{ \begin{array}{c} i_{t}=\sigma \,\left(W_{i}\,\left[e_{t+1},g_{t-1},c_{t-1}\right]+b_{i}\right)\;\\ f_{t}=\sigma \,\left(W_{f}\,\left[e_{t+1},g_{t-1},c_{t-1}\right]+b_{f}\right)\;\\ o_{t}=\sigma \,\left(W_{o}\,\left[e_{t+1},g_{t-1},c_{t-1}\right]+b_{o}\right)\;\\ c_{t}=f_{t}\,c_{t-1}+i_{t}\,\tanh\left(W_{c}\,\left[e_{t+1},g_{t-1}\right]+b_{c}\right)\\ g_{t+1}=o_{t}\,t\,a\,n\,h\,\left(c_{t}\right)\; \end{array}\right.$$

(15) $$m_{t+1}=\mathrm{Monotonic}\,\_\,\mathrm{Attention}\,\left(e_{t+1},e_{t+1},g_{t+1}\right)$$

(16) $$s_{t+1}=L\,2\,\_\,\mathrm{normalize}\,\left(W_{h,\mathrm{KS}}\,m_{t+1}+b_{h,\mathrm{KS}}\right)$$

#### Knowledge Status Query

Do the dot product operation on the dimensional knowledge state vector *KS*_*t*_ and the dimensional question vector *S*_*t+1*_ obtained by the knowledge state encoder and the item encoder, respectively, and then input the result into the sigmoid activation function to obtain the final prediction of the probability *p*_*t+1*_ that the learner answers the next question correctly. Calculated as follows:


(17) $$y_{t+1}={\mathrm{KS}}_{t}\cdot S_{t+1}$$

(18) $$p_{t+1}=\sigma \,\left(y_{t+1}\right)$$

The dot product of knowledge state vector and question vector conforms to the process of real world middle school learners answering questions based on their own knowledge state (Lee and Yeung, 2019), which makes the model more explanatory.

### Optimization

We use the backpropagation algorithm to train the network model, and update the model parameters by minimizing the cross entropy loss of the prediction probability and the labeled result. At each time step *t*, calculate the cross entropy loss result of a single learner, and sum the *t* = 1,⋯,*T*−1 loss of all learners to get the total loss. The specific calculation process is:


(19) $$\ell \,\left(\theta_{\mathrm{model}}|r_{t+1}^{i},p_{t+1}^{i}\right)=-\left[r_{t+1}^{i}\,{\mathrm{logp}}_{t+1}^{i}+\left(1-r_{t+1}^{i}\right)\,\log\,\left(1-p_{t+1}^{i}\right)\right]$$

(20) $$ℒ\,\left(\theta_{\mathrm{model}}|r_{2:t+1},p_{2:t+1}\right)=\sum_{i}\sum_{t=1}^{T-1}\ell \,\left(\theta_{\mathrm{model}}|r_{t+1}^{i},p_{t+1}^{i}\right)$$

## Experiments

In this section, we first introduce the details of the dataset, experimental parameter settings and evaluation indicators, and then show the performance of this model and other models in 4 real-world online education datasets. Finally, we use ablation experiments to further verify the effectiveness of the Rasch model-based embedding, monotonic attention mechanism and question context-aware representation.

### Datasets

We used four publicly available real online education datasets to evaluate the model, namely ASSISTments2009, ASSISTments2015, ASSISTments2017^1^, and Statics2011^2^. The ASSISTments datasets are collected from the ASSISTments online tutoring platform. And the ASSISTments2009 dataset has been the accepted standard dataset of the KT method for the past 10 years. The Statics2011 dataset was collected from a university-level statics engineering course. In all datasets, the preprocessing steps in this paper follow a series of standards in Ghosh et al. (2020). In Table 1, we list the number of learners, KCs (i.e., concepts, knowledge points), questions, and learner interaction tuples. In these datasets, only the ASSISTments2009 and ASSISTments2017 datasets contain question IDs. Therefore, the model based on the Rasch model embedding is only applicable to these two datasets.

**TABLE 1:** Statistics of dataset.

Dataset	learners	KCs	Questions	Responses
ASSISTments2009	4,151	110	16,891	325,637
ASSISTments2015	19,840	100	–	683,801
ASSISTments2017	1,709	102	3,162	942,816
Statics2011	333	1,223	–	189,297

### Experimental Setup and Evaluation Index

We use the five-fold cross-validation method to start the experiment based on PyTorch version 1.2.0. The division of all datasets is consistent with Ghosh et al. (2020), 20% is used as the test set, 20% is used as the validation set, and 60% is used as the training set. And we use the grid search method on the validation set to determine the optimal parameters. We use {10^−6^, 10^−5^, 10^−4^, 10^−3^, 10^−2^}, {64, 128, 256, 512}, {64, 128, 256, 512}, {0, 0.05, 0.1, 0.15, 0.2, 0.25}, and {32, 64, 128, 256, 512} as values of the learning rate, the input embedding dimension, the hidden state dimension of LSTM, the dropout rate for the LSTM network, and the dimension of knowledge state vector and question vector, respectively. Finally, we set the maximum number of epochs to 300, the default optimizer to Adam, the learning rate to 10^−4^, batch size to 32, the input embedding dimension to 128, the dimension of the LSTM hidden layer to 128, the dropout rate to 0.1, the dimension of knowledge state vector and question vector to 128.

With reference to most of the KT research work, we use the area under the curve (AUC) as an evaluation model to predict the performance of the learner’s next interaction. The higher the AUC, the better the model’s predictive performance.

### Experimental Results and Analysis

#### Comparative Experiment

On four educational datasets, the CAKQN model proposed in our paper is compared with several common traditional network KT model representatives including IRT+ (Pardos and Heffernan, 2011), BKT+ (Yudelson et al., 2013) and neural network representative baseline models, including DKT (Piech et al., 2015), DKVMN (Zhang et al., 2017), KQN (Lee and Yeung, 2019), SAKT (Pandey and Karypis, 2019), AKT (Ghosh et al., 2020), the experimental results are shown in Table 2. Note that best models are bold, the results with * are form other paper.

**TABLE 2:** The predicted results of different methods on knowledge tracing.

Model	AUC (%)			
	
	ASSISTments2009	ASSISTments2015	ASSISTments2017	Statics2011
IRT+	77.40*	–	–	–
BKT+	69*	–		75*
DKT	80.53 ± 0.2*	72.52 ± 0.1*	72.63 ± 0.1*	80.20 ± 0.2*
DKVMN	81.57 ± 0.1*	72.68 ± 0.1*	70.73 ± 0.1*	82.84 ± 0.1*
KQN	82.32 ± 0.05*	73.40 ± 0.02*	73.33 ± 0.03*	83.20 ± 0.05*
SAKT	84.8*	85.4*	72.12*	85.3*
AKT-NR	81.69 ± 0.004*	78.28 ± 0.002*	72.82 ± 0.003*	82.65 ± 0.004*
AKT-R	83.46 ± 0.003*	–	77.02 ± 0.002*	–
CAKQN-R	**87.04 ± 0.004**	–	**79.33** ± **0.002**	–
CAKQN-NR	85.54 ± 0.003	**88.88 ± 0.004**	76.45 ± 0.003	**85.43** ± **0.001**

The symbol * means the result is from other paper. The best results are shown in bold.

Table 2 lists the performance of all KT methods across all datasets for predicting future learner responses. CAKQN-R and CAKQN-NR represent variants of the CAKQN model with and without the embedding based on the Rasch model, respectively. Similarly, AKT-R and AKT-NR represent variants of the AKT model with and without the embedded Rasch model in Ghosh et al. (2020), respectively. The experimental results show that the CAKQN-R model proposed in this paper is better than the existing model, and its AUC value is 2.945% higher than the existing optimal model AKT-R on average. Note that IRT+ and BKT+ have the lowest prediction performance on the four datasets compared to the neural network representing the four datasets. This indicates that both methods rely on experts to label KC, and the model cannot capture more information like deep neural networks. In the DLKT model, the average prediction performance of the KQN model on the four datasets is significantly improved compared to DKT and DKVMAN. This is because the KQN model is more explanatory in terms of learner knowledge interaction. And CAKQN-R and CAKQN-NR, which also use dot products to represent the interaction process between learner knowledge and questions, have achieved better performance on all datasets. This is related to its different network structure, the monotonic attention mechanism introduced and the embedding based on the Rasch model. Taking a closer look, the SAKT, AKT, and CAKQN models that introduce the attention mechanism and its variants have achieved better results than the general DLKT models such as DKT, DKVMN, and KQN. Because the attention mechanism can link the KC at the next time step with the related KC in the learner’s past interaction sequence, the DLKT model with the attention mechanism can more accurately describe the knowledge state of each learner, thereby improving the performance of the model. Among them, the CAKQN-R model achieved better results than other DLKT models with attention mechanisms on the two ASSISTments datasets with question IDs. This proves that the CAKQN-R model can dig more complex features such as forgetting behavior in learner interaction sequences, obtain more accurate learner knowledge status and improve the prediction effect. Comparing the CAKQN-NR and AKT-NR models with the same monotonic attention mechanism, CAKQN-NR model proposed in this paper uses the network structure of LSTM+monotonic attention mechanism to retain the ability of the model to model the sequence, which can not only ensure that the original learner’s historical learning interaction sequence is not damaged on the time scale, but also extract complex features of learners such as forgetting behavior. More importantly, it also provides a more interpretable interaction process between learner knowledge and questions, which contributes to a better prediction effect than AKT-R. Finally, comparing CAKQN-R and CAKQN-NR, we found that CAKQN-R has better prediction performance on both datasets. This proves that the embedding based on the Rasch model can capture the characteristics of individual differences between different questions of the same KC and the personal abilities of learners, and obtain more accurate personalized representations of learner interaction tuples and questions, thereby improving the performance of the model.

#### Ablation Experiment

In order to further verify the three key innovations in the CAKQN model: context-aware representation of question vectors, monotonic attention mechanism, and embedding based on the Rasch model, three additional ablation experiments were carried out in this paper. The first experiment is the comparison of CAKQN-R, CAKQN-NR and its variants CAKQN^raw^-R and CAKQN^raw^-NR. The structure of CAKQN^raw^-R and CAKQN^raw^-NR Question Encoder is the same as the KQN model. It uses a multi-layer perceptron (MLP) to directly input the question embedding to obtain the question vector, the number of hidden layers is 1 and the dimension is 128. The second experiment is to compare CAKQN-R, CAKQN-NR, SAKT models and two variants CAKQN-R*^nl^* and CAKQN-NR*^nl^* without monotonic attention mechanism. The tow variants use ordinary dot product attention to capture the time dependence in the learner’s response data. The last one is the experiment is a comparison between CAKQN-R and variant CAKQN-IRT. The CAKQN-IRT model is based on the DIRT framework proposed in Cheng et al. (2019). Specifically, the *Knowledge State Encoder* and *Question Encoder* components used in the CAKQN-IRT model are the same as CAKQN-R, but the difference is that CAKQN-IRT uses direct embedding instead of Rasch embedding. The *Knowledge State Encoder* component of CAKQN-IRT obtains the learners’ ability θ, one *Question Encoder* component inputs the question and KC embedding to obtain the distinction of the question α_*j*_, and the other exactly the same *Question Encoder* component inputs the question embedding to obtain the difficulty of the question β_*j*_. Finally, the obtained parameters are substituted into the two-parameter IRT model formula in section “Traditional Knowledge Tracking Methods” for prediction.

Table 3 shows the results of the first ablation experiment based on the context-aware representation of the question vector. In all datasets, CAKQN-R and CAKQN-NR are better than CAKQN^raw^-R and CAKQN^raw^-NR. These results show that our context-aware representation of the question is effective in summarizing the relationship between the question at the next time step and the historical question.

**TABLE 3:** Experimental comparison between CAKQN and variant that do not use contextual aware question and response representations.

Model	AUC (%)			
	
	ASSISTments2009	ASSISTments2015	ASSISTments2017	Statics2011
CAKQN^raw^-NR	84.49 ± 0.004	85.31 ± 0.004	74.84 ± 0.002	85.13 ± 0.001
CAKQN-NR	**85.54 ± 0.003**	**88.88 ± 0.004**	**76.45 ± 0.003**	**85.43 ± 0.001**
CAKQN^raw^-R	86.12 ± 0.004	–	77.14 ± 0.003	–
CAKQN-R	**87.04 ± 0.004**	–	**79.33 ± 0.002**	–

*The best results are shown in bold.*

Table 4 shows the results of the second ablation experiment of the monotonic attention mechanism. On all datasets, CAKQN-NR is significantly better than other attention mechanisms, including SAKT. In the case of both using Rasch-based model embedding, CAKQN-R still achieves better results than CAKQN-R*^nl^* on the two datasets. The reason for this is that it is different from the common language tasks with strong long-distance dependence between words. The dependence of future learner performance on the past is restricted to a much shorter time window for their forgetting behaviors. Therefore, the monotonic attention mechanism with exponential decay when calculating the attention weight can effectively capture the short-term dependence on the past on the time scale to simulate the forgetting behavior of learners in the learning process.

**TABLE 4:** Experimental comparison between CAKQN and variants with other attention mechanism.

Model	AUC (%)			
	
	ASSISTments2009	ASSISTments2015	ASSISTments2017	Statics2011
SAKT	84.8*	85.4*	72.12*	85.3*
CAKQN-NR*^nl^*	84.01 ± 0.005	80.52 ± 0.011	71.84 ± 0.004	83.89 ± 0.001
CAKQN-NR	**85.54 ± 0.003**	**88.88 ± 0.004**	**76.45 ± 0.003**	**85.43 ± 0.001**
CAKQN-R*^nl^*	85.52 **±** 0.004	–	75.44 ± 0.003	–
CAKQN-R	**87.04 ± 0.004**	–	**79.33 ± 0.002**	–

The symbol * means the result is from other paper. The best results are shown in bold.

Table 5 shows the results of the third ablation experiment based on the embedding of the Rasch model. Both models are only tested on the two ASSISTments datasets where the question ID in the dataset is available. On these two datasets, CAKQN-R is significantly better than CAKQN-IRT in the predictive ability of the model. This shows that although CAKQN-IRT incorporates a more complex two-parameter IRT model, CAKQN-R has achieved better results with a simpler model structure. This also confirms that CAKQN-R has more advantages in the knowledge interaction process represented by the dot product calculation in the knowledge query component.

**TABLE 5:** Experimental comparison between CAKQN and CAKQN-IRT.

Model	AUC (%)	
	
	ASSISTments2009	ASSISTments2017
CAKQN-IRT	84.43 ± 0.015	75.33 ± 0.020
CAKQN-R	**87.04 ± 0.004**	**79.33 ± 0.002**

*The best results are shown in bold.*

#### Visualization of Knowledge Tracking

**FIGURE 3:**
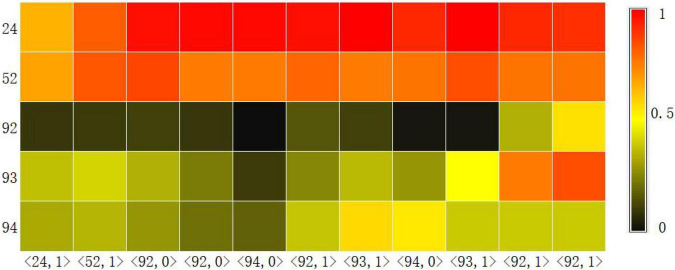
The knowledge level output result of CAKQN-R*^nl^* on the ASSISTments2009 dataset.

**FIGURE 4:**
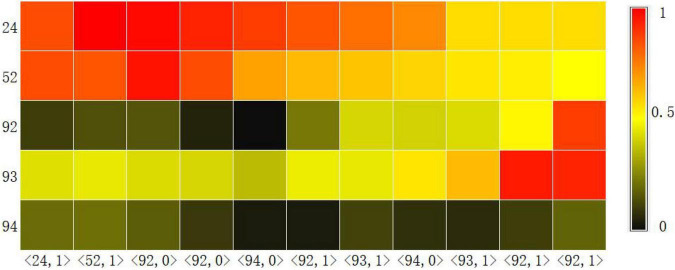
The knowledge level output result of CAKQN-R on the ASSISTments2009 dataset.

Another basic task of knowledge tracking is to show learners’ mastery of each knowledge point in real time. Therefore, we visualized the probability of learners answering correctly at each knowledge point at each time step through the Knowledge Query component. We intercepted the learning records of a learner in the dataset ASSISTments2009 over a period of time, and used the CAKQN-R*^nl^* and CAKQN-R model models to track the changes in learners’ mastery of 5 knowledge points, as shown in Figures 3, 4. The horizontal axis in the figure represents the interception of the learner’s 11 time steps of learning history. The in the tuple represents the learner’s KC (knowledge points), represents the learner’s answer. The vertical axis represents the 5 knowledge points tracked by the model.

From the visualization results, it can be seen that at the first time step, after the learners answered the exercises containing knowledge points 24 correctly, the tracking results of CAKQN-R*^nl^* and CAKQN-R on the learners’ knowledge points 24 have been improved (the probability of correct answers increases). The results indicate that the CAKQN-R*^nl^* model and the CAKQN-R model will update the mastery of the corresponding knowledge points accordingly after obtaining the learner’s historical answer results. In Figures 3, 4, within ten time steps after the learner correctly answered the exercises containing knowledge point 24 at the first time step, CAKQN-R*^nl^* did not update the learner’s mastery of knowledge point 24, while CAKQN-R showed that the degree of learner’s mastery of knowledge point 24 has been declining. It can be seen that the CAKQN-R*^nl^* model does not consider the learner’s forgetting behavior during the learning period, and the CAKQN-R model fits the learner’s actual forgetting behavior during the learning period after introducing the multiplicative exponential decay term. The above results show that both the CAKQN-R model and the CAKQN-R*^nl^* model can model the learning process of learners’ knowledge status over time. However, the CAKQN-R*^nl^* model cannot model the forgetting behavior of learners, while the CAKQN-R model can model the forgetting behavior of learners, and more accurately track learners’ mastery of various knowledge points in real time.

## Conclusion

Real-time assessment of learners’ online learning knowledge level helps to monitor learners’ own cognitive status, adjust learning strategies, and improve the quality of online learning. As for four real online education datasets, this paper proposes a CAKQN model based on Rasch model embedding. It uses the vector dot product to describe the interaction process between the learner’s knowledge state and the question, and uses the network structure of LSTM + monotonic attention mechanism to capture the question and the learner’s personalized contextual representation. Compared with most other knowledge tracking models, it can not only track learners’ knowledge status in real time, but also model learners’ forgetting behavior.

However, the method presented in this paper has several limitations.

(1) CAKQN uses binary variables to represent the answer to the question as same as other KT methods. This way is not suitable for subjective questions with continuous score distribution. Wang et al. (2017) and Swamy et al. (2018). provide a new way to model subjective questions, they used continuous snapshots of the learner’s answers as an indicator of the answer when dealing with learners’ programming data. Modeling subjective topics will be the direction of future research.

(2) The adaptive capacity of the model needs to be improved. CAKQN is a supervised training method like other deep knowledge tracking methods, so the predictive ability of the model is dependent on the effect of training on the current dataset. If you are faced with small data sets or other domain datasets, the performance of the model may be poor (Wang Y. et al., 2021).

(3) Like most other KT methods, our method is based on the learner’s historical practice record modeling, and involves too few features. In fact, the learning process is very complex, involving many other features such as the text of the question, the learning rate of the student, and the positive/negative emotions that the student generates during the learning process. At present, with the rapid development of technologies such as intelligent perception, wearable devices, and the Internet of Things, multi-modal learning analysis will become a new trend driving intelligent education research (Wang Z. et al., 2021). Under this trend, knowledge tracking will surpass a single behavior modality and gradually develop into a learner model driven by the fusion of multimodal data such as behavior, psychology, and physiology.

## Data Availability Statement

The original contributions presented in this study are included in the article/supplementary material, further inquiries can be directed to the corresponding author/s.

## Author Contributions

YC: writing – review and editing, supervision, resources, and conceptualization. GW: methodology, software, validation, and writing – original draft. HZ: visualization and writing – review and editing. PL: data curation. ZC: formal analysis. All authors contributed to the article and approved the submitted version.

## Conflict of Interest

The authors declare that the research was conducted in the absence of any commercial or financial relationships that could be construed as a potential conflict of interest.

## Publisher’s Note

All claims expressed in this article are solely those of the authors and do not necessarily represent those of their affiliated organizations, or those of the publisher, the editors and the reviewers. Any product that may be evaluated in this article, or claim that may be made by its manufacturer, is not guaranteed or endorsed by the publisher.

## Footnotes

1 The ASSISTments datasets are retrieved from https://sites.google.com/site/assistmentsdata/home and https://sites.google.com/view/assistmentsdatamining/.

2 The Statics2011 dataset is retrieved from https://pslcdatashop.web.cmu.edu/DatasetInfo?datasetId=507.
